# Electro-Gilding

**Published:** 1907-09-15

**Authors:** J. Tenney Barker


### ELECTRO-GILDING.

BY J. TENNEY BARKER, D. D. S.

Electro-gilding, more commonly called gold-plating, as practiced in the arts: The question you probably will ask, is—why interesting to us? I will tell you, knowing you all to be honest and conscientious men; if you were not, I should not treat upon this subject. To commence with, any piece of mechanical work composed of several carats of gold and gold solder will when finished contain several shades of gold. If electro-plated with fine gold, it will present a more harmonious and finished piece of work and one that you will be proud of. Again, in constructing regulating appliances, it is much more artistic to cover the base metals usually used in constructing such with a heavy coating of pure gold.

Mouth mirrors and foil carriers, as well as many other instruments, may be made to present a much more attractive appearance. For several years it has been my practice to treat a new mouth mirror to a good healthy plate, thus, always knowing a new mirror from an old one at a glance.

It would consume so much time enumerating the several uses electro-plating may be used for, that I will leave to your judgment when and where to take advantage of it, and proceed to describe the apparatus, etc., necessary to accomplish the best results, as well as the preparation of articles to be gilded.

First: One must possess a good battery. Formerly it was thought that a "Smee battery" only could be successfully used; however, for the past few years I have used the Samson batteries, No. 2. They are long lived, and require no care when not in use, except to keep the solution up to the paraffin line along the neck of the glass jar.

Second: It is absolutely necessary that a glass or porcelain cup be used as a receptacle for the gold solution, and better still, a double porcelain lined dish.

Third: We must now secure a piece of fine gold to be used as an anode; a piece one or two inches long, and three-fourths of an inch wide, will do. However, my practice is now to roll the gold for the anode out into a strip, gauge thirty-two, and double it upon itself several times, then punch a hole through one end and connect it to a wire running to and connected with the positive pole of the battery, which is the carbon of the "Samson cell"—the zinc always being the negative pole.

For the benefit of those who prefer to prepare their own gold solution, I will give the following directions:

The best and cheapest method of making up the solution is known as "the battery process." It saves all expense of acids and labor of precipitation and washing necessary in preparing it from the chloride of gold. In preparing one gallon of gold solution, dissolve four ounces of cyanide of potassium in one gallon of water, and heat to 150° F. Next, take a small porous cell and fill it with this cyanide solution and place it inside of the receptacle containing the balance of the gallon solution, the two solutions to be on a level: then connect either a small piece of iron or copper with a wire attached to the zinc of battery; immersing the iron or copper plate in the small porous cell. A piece of pure gold is connected to the carbon of battery and placed in the large jar of solution, facing the plate in the porous cell. The whole to remain in action until the gold, which is to be taken out from time to time and weighed, has lost the quantity required in solution, which should be one dwt. to the quart. The solution in the porous cell may be thrown away, as there will not be any gold there, except the action has been continued a long time, and if any, it will not amount to enough to pay to save it.

However, it is much more satisfactory to purchase your gold solution from some reputable goldplater, the cost of same being about $1.50 per pint and if your battery is kept in perfect condition with a good anode, it will not become exhausted.

Every article should be carefully polished and finished with a perfect surface, then carefully washed in hot water with soap and ammonia, rinsed in cold water, and then washed in a strong hot or warm solution of carbonate of soda and water. After washing, rinse well in cold water, and immediately hang upon the wire connected with the negative pole of the battery and suspended from all objects, being careful that nothing comes in contact with it; for the least particle of foreign matter will affect the plating of the same, and destroy the final result.

If you have carefully followed me, we are now ready to commence to deposit our gold. Place the gold solution in a cup or other vessel that is a non-conductor, then place this cup in a pan of water—the water reaching to a level with the gold solution—placing the whole on a suitable stove, and gradually heat to a point just before the boiling point, then immerse the article to be plated, connected with the zinc or negative pole of battery in the solution and the anode or strip of pure gold connected with the positive pole or carbon of battery, being very careful not to short circuit the battery by allowing both poles coming in contact, then gently agitate the cathode or articles to be plated.

I want to state right here that the solution should be in such quantity that it will more than cover the article, for, if it does not, when the cathode is agitated, if it strikes the receptacle holding the solution, there is a liability of scratching the same. As soon as a blush of gold is deposited on the cathode, remove, rinse and scratch brush with a soft brass wire brush wheel, then wash as before and return to bath, allowing ample time for a good deposit of gold—from six to ten minutes is sufficient, ordinarily. If one wants to be exact, weigh the piece to be plated before immersing in solution, and then from time to time return it to the scales, and weigh until you have the desired amount of gold deposited. As the gold solution evaporates by being kept hot, distilled water must be added from time to time to retain the solution. This water should always be added to the completion of the operation of gilding, for if it is added just before, the solution will not give as good results. Much depends upon the heat of the solution and state of the battery to obtain the best results; therefore it is necessary to pay the strictest attention to both, as well as to the size and loss to the anode, or the plate of pure gold. This should at all times have a surface equivalent to the article to be plated. It will not hurt if it is larger. If the gold pole has a film or crust upon it when taken from the solution it is a certain indication that the solution is deficient in cyanide of potassium, and a small amount should be added. If the pole comes out clear it is all right. Care must be taken to distinguish the crust—which is occasionally dark green or black—from a black appearance which the gold pole will take when it is very small, in comparison with the article being plated, and which is caused by the evolution of gas. In this case an addition of cyanide of potassium would increase the evil. The black appearance, coming from a tendency to the escape of gas, has a slimy feeling and look. This generally takes place when the solution is nearly exhausted of gold. In regulating the color of the plate a very rich dark shade may be made by adding ammoniuret of gold to the solution, just as the articles are being put into the solution. If a bright, clear yellow color is desired—that will not require scratch brushing—add a small quantity of caustic soda. However, all articles, regardless of color desired, should come from the solution with a dark yellow color, approaching to a brown; this when scratch brushed will yield a rich, deep gold. If the color is blackish it ought never to be finished, for it will never brush or burnish a good color. If the battery is too strong and gas is given off from the article, the color will be black; and if the solution is too cold or the battery weak, the gold will be light colored. All work should be burnished to secure the best results, and anyone may soon learn how to accomplish this with proper tools to secure the ideal results.—*Dental Brief.*

